# Dentate-nucleus gadolinium deposition on magnetic resonance imaging: ultrasonographic and clinical correlates in multiple sclerosis patients

**DOI:** 10.1007/s10072-021-05702-4

**Published:** 2021-11-04

**Authors:** Isabelle Kühn, Henning Maschke, Annette Großmann, Karlheinz Hauenstein, Marc-André Weber, Uwe K. Zettl, Alexander Storch, Uwe Walter

**Affiliations:** 1 Department of Neurology, Rostock University Medical Center, University of Rostock, Gehlsheimer Str. 20, 18147 Rostock, Germany; 2 Department of Dermatology and Venereology, University Hospital Hamburg-Eppendorf (UKE), Hamburg, Germany; 3 Institute of Diagnostic and Interventional Radiology, Paediatric Radiology and Neuroradiology, Rostock University Medical Center, Rostock, Germany; 4 Department of Radiology and Neuroradiology, Asklepios Hospital Barmbek, Hamburg, Germany; 5 German Center for Neurodegenerative Diseases (DZNE), Research Site Rostock, Rostock, Germany

**Keywords:** Dentate nucleus, Gadolinium, MRI contrast media, Multiple sclerosis, Transcranial ultrasonography

## Abstract

**Objective:**

The objective of this study is to find out whether gadolinium accumulation in the dentate nucleus (DN) after repeated gadolinium-based contrast agent (GBCA) administration in multiple sclerosis (MS) patients is related to tissue alteration detectable on transcranial ultrasound.

**Methods:**

In this case–control study, 34 patients (17 with, and 17 age-, sex-, MS severity-, and duration-matched participants without visually rated DN T1-hyperintensity) who had received 2–28 (mean, 11 ± 7) consecutive 1.5-Tesla MRI examinations with application of linear GBCA were included. Real-time MRI-ultrasound fusion imaging was applied, exactly superimposing the DN identified on MRI to calculate its corresponding echo-intensity on digitized ultrasound image analysis. In addition, cerebellar ataxia and cognitive performance were assessed. Correlation analyses were adjusted for age, MS duration, MS severity, and time between MRI scans.

**Results:**

DN-to-pons T1-signal intensity-ratios (DPSIR) were larger in patients with visually rated DN T1-hyperintensity compared to those without (1.16 ± 0.10 vs 1.09 ± 0.06; *p* = 0.01). In the combined group, DPSIR correlated with the cumulative linear-GBCA dose (*r* = 0.49, *p* = 0.003), as did the DPSIR change on last versus first MRI (*r* = 0.59, *p* = 0.003). Neither DPSIR nor globus pallidus internus-to-thalamus T1-signal intensity-ratios were related to echo-intensity of corresponding ROI’s. DPSIR correlated with the dysarthria (*r* = 0.57, *p* = 0.001), but no other, subscore of the International Cooperative Ataxia Rating Scale, and no other clinical score.

**Conclusions:**

DN gadolinium accumulation is not associated with trace metal accumulation, calcification, or other tissue alteration detectable on ultrasound. A possible mild effect of DN gadolinium accumulation on cerebellar speech function in MS patients, suggested by present data, needs to be validated in larger study samples.

**Supplementary Information:**

The online version contains supplementary material available at 10.1007/s10072-021-05702-4.

## Introduction

Gadolinium (Gd)-based contrast agents (GBCAs) are widely used to improve diagnostic accuracy of magnetic resonance imaging (MRI). Especially, in patients with multiple sclerosis (MS), repeated GBCA-enhanced MRI of the central nervous system is recommended at the time of diagnosis, during follow-up, while on- or off-therapy, and when intercurrent diseases and side effects such as progressive multifocal leukoencephalopathy are suspected [1–3]. Gd-enhancement in cerebral or spinal MS lesions indicates disruption of the blood–brain barrier which is a surrogate for recent and active inflammation. Since 2013, a number of studies demonstrated Gd retention, apart from MS lesions, in the cerebellar dentate nucleus (DN) and, to a much lesser degree, the basal ganglia as a result of repeated intravenous GBCA injections [4–10]. It has been shown that linear GBCAs are more likely to accumulate than macrocyclic agents [10–16] and that the chemically less stable linear non-ionic (e.g., gadodiamide (Gd-DA, Omniscan®)) are more likely to accumulate than the more stable linear ionic GBCAs (e.g., gadopentetate dimeglumine (Gd-DTPA, Magnevist®)) [17, 18].

Classically, Gd retention in the DN is visualized on unenhanced T1-weighted MRI and is measured as a relative signal intensity increase in the respective DN-to-comparator ratio [4–12], preferably the DN-to-pons signal intensity ratio (DPSIR) [19]. On the other hand, transcranial brain sonography (TCS) sensitively detected tissue alterations caused by the deposition of trace metals, neurodegeneration, and calcification of deep brain structures, including the DN [20–24]. So far, it is unclear whether chronic gadolinium deposition in the brain may be associated with changes of echogenicity on TCS or neurological or neuropsychological deficits. Here we studied 34 MS patients who had received 2–28 (median, 11) MRI scans with application of linear ionic and nonionic GBCAs. The main goal of the present study was to test the hypothesis that the change of DPSIR corresponds to change of DN echogenicity on TCS. In addition, we analyzed whether an increase in the DPSIR after multiple GBCA administrations may be related to signs of cerebellar dysfunction.

## Materials and methods

### Study design

From our research database, we retrospectively identified MS patients who participated in long-term longitudinal studies and were repeatedly administered the linear non-ionic Gd-DA (Omniscan®) and/or the linear ionic Gd-DTPA (Magnevist®) between 1999 and 2016, each at a dosage of 0.2 mL per kilogram body weight with a concentration of 0.5 mmol/mL (0.1 mmol per kilogram body weight per dosage) according to the manufacturers’ instructions. The patients were classified into two groups according to the presence (T1h^+^) or absence (T1h^−^) of visible T1-hyperintensity of DN on at least one of the three most recent MRI scans confirmed by a senior radiologist (A.G.) and were selected for this study into the groups T1h^+^ and T1h^−^ in an age-, sex-, MS severity-, and duration-matched manner (case–control design). The change of DPSIR on unenhanced T1-weighted spin echo sequences between the first and the last scan was assessed independently by a qualified radiologist. The exposures were the total number of linear GBCA administrations, the cumulative dose of linear GBCA per kilogram body weight, and the cumulative dose of either the ionic Gd-DTPA or the non-ionic Gd-DA per kilogram body weight. In the prospective part of this study, performed between April 2017 and June 2018, real-time TCS-MRI fusion imaging was applied to exactly superimpose the DN identified on recently acquired MRI and the corresponding region on TCS, in order to assess the echogenicity of DN in the same ROI on digitized image analysis. Clinical assessments in the prospective part of the study included the scoring of cerebellar ataxia and cognitive performance. Age, MS duration, MS severity assessed on the Expanded Disability Status Scale (EDSS), and the time between MRI scans were considered as potential variables of relevance. The ethics committee at the Rostock University Medical Center approved the study (identifier: A 2017–0026). All patients provided written informed consent for participation in this study.

### Participants

A total of 34 participants (17 per group T1h^+^ and T1h^−^), who had received between two and 28 (mean, 11 ± 7) consecutive MRI examinations with application of Gd-DTPA or Gd-DA, were included in our study. The patients did not receive any dose of GBCA prior to the first MRI scan used to calculate the DPSIR change. The first scan (if used for calculation of DPSIR change) and the most recent scan (the last one not necessarily with application of GBCA) had had to be carried out on a 1.5-T scanner (Magnetom Avanto; Siemens) according to standardized protocols as part of clinical studies performed at the Department of Neurology, Section of Neuroimmunology, at the Rostock University Medical Center, without any change of field strength during the follow-up. Exclusion criteria were high infratentorial lesion load affecting the DN and inability to give informed consent. All patients had a definite diagnosis of MS according to panel criteria [25]. None of the participants had known renal impairment. Clinical and demographic data, total number and timing of scans with administration of linear GBCA, and the type of GBCA administered were retrieved from the research database.

### MR image acquisition and processing

The brain MRI scans analyzed in this study were performed on a 1.5-T scanner (Magnetom Avanto; Siemens Healthineers) by using a device-specific 12-channel head coil. Parameters for T1-weighted imaging were as follows: distance factor, 10%; field of view read/field of view phase, 230 mm/75%; slice thickness, 3 mm; repetition time, 480 ms; echo time, 17 ms; voxel size, 1,2 × 0,6 × 5 mm. The applied GBCA dose depended on the patient’s body weight (see above).

The review and quantitative analysis of unenhanced T1-weighted images were done by a radiologist (H.M.) with 5 years’ experience in brain MRI who could in uncertain cases consult a board-certified neuroradiologist (A.G.) with 25 years’ experience in brain MRI, both being blinded to clinical and TCS data of the patients. The T1-signal intensity increase was assessed according to the recommendations of the European Gadolinium Retention Evaluation Consortium and the European Society of Neuroradiology [19]. For measuring the signal intensity in unenhanced T1-weighted images on the PACS workstation (IMPAX 6; Agfa HealthCare), a region of interest was manually drawn around the dentate nucleus and in the pons. T2-weighted sequences were available to help localize the nuclei. The regions of interest allowed the measurement of the averaged signal intensity in an individually defined area. By dividing the averaged signal intensity of the DN by the averaged signal intensity of the pons, the DPSIR was determined (average value of bilateral DN). Ultimately, changes in DPSIR over time were calculated by subtracting the DPSIR at the first assessable MRI from that at the last MRI. Since digitized MRI data sets were present in our database since July 2005, calculation of DPSIR changes was restricted to MRI scans registered since then. Analogously, the globus pallidus internus (GPi)-to-thalamus signal intensity ratio (GTSIR) and GTSIR differences were calculated.

### TCS image acquisition and processing

For MRI-TCS fusion imaging, a high-end ultrasound system (MyLabTwice; Esaote S.p.A.) equipped with a 2.5-MHz phased-array transducer (PA240) and fusion imaging technology (Virtual Navigator) was applied. The ultrasound system settings were as follows: view, 3; size of aperture, 89°; dynamic range, 6; dynamic compression, 2; persist, 7; enhance, 3; density, 2; focuses, 1; SView, off; colorize, 0; gray map, 5. The DICOM volume dataset from pre-acquired MRI examination was loaded into the ultrasound system. Then the image registration and fine-tuning procedures were carried out to exactly superimpose the MRI and TCS images as described earlier in detail [26, 27].

Once real-time TCS examination was being performed, the MRI volume was also being scrolled automatically by the system, showing the same planes. This allowed then for the clear delineation of the ROIs of DN and GPi, being evident on MRI, also in the corresponding plane on TCS (see Supplementary Material 1). The TCS images of DN and GPi were saved for digitized analysis. To quantify the echo-intensity of DN and GPi, off-line digitized image analysis was performed using a validated MATLAB-based software tool [27]. This tool pre-assesses the overall image quality before starting the analysis of distinct structures, yielding a normalized echo-intensity measure of the target structure in the referring ROI. Mean values of bilateral DN, and of bilateral GPi, echo-intensity per patient were used for further analyses.

### Clinical assessments

The clinical assessments were carried out by an investigator (I.K.) blinded to MRI and TCS imaging data. The severity of cerebellar ataxia was assessed on the International Cooperative Ataxia Rating Scale (ICARS), which has been validated for mild to moderate MS [28, 29]. Since the DN is involved also in cognitive performance [30, 31], we additionally applied the Digit Symbol Substitution Test (DSST) and the 3-s Paced Auditory Serial Addition Test (PASAT-3) [32, 33]. The DSST is a widely used measure of psychomotor speed, sustained attention, visual-spatial skills, and set-shifting [32]. The DSST version used here consists of four rows containing a total of 93 small blank squares, each of which is paired with a randomly assigned number from 1 to 9. The number of correct responses achieved within 90 s was summed and recorded as the score. The PASAT-3 is a measure of sustained attention and speed of information processing [33]. The PASAT-3 test scores were normalized to *z* scores on the basis of normative data.

### Statistical analyses

For comparison of means, the *t* test for independent samples was used, and for comparison of non-normally distributed data the Mann–Whitney *U* test. Categorical data were analyzed by Fisher’s exact test. The Pearson correlation test was used to compare DPSIR and GTSIR measures with cumulative dose of linear GBCA, cumulative dose of Gd-DTPA, cumulative dose of Gd-DA, echo-intensity measures, and clinical scores (ICARS, DSST, PASAT-3). The correlation analyses were adjusted for age, MS duration, EDSS, and latency between first and last MRI as potential confounders. Since seven variables were compared, a Bonferroni correction was employed, with the significance level set at *p* ≤ 0.007 for the correlation analyses. When the absolute effect of GBCA administration on T1-intensity was tested, a one-tail *p* value was used assuming a positive association between GBCA administration and T1-signal intensity increase. In all other cases, a two-tail *p* value was used.

## Results

Altogether, 34 patients were studied: 17 without (T1h^−^) and 17 age-, sex-, MS severity-, and duration-matched patients with (T1h^+^) visible T1-hyperintensity of DN. Calculation of DPSIR and GTSIR differences was possible for 23 patients (68%; T1h^−^, *n* = 9; T1h^+^, *n* = 14); in the remaining eleven cases, the digitized data of the first MRI were either not available (*n* = 6) or did not fulfill the quality criteria for valid T1 intensity ratio calculation (*n* = 5). Twenty-one patients (62%; T1h^−^, *n* = 10; T1h^+^, *n* = 11) agreed with participation in the elaborate MRI-TCS fusion imaging procedure. Demographic, clinical, MRI, and MRI-TCS fusion imaging data are summarized in Table 1. Even though the means of the objectively quantified DPSIR measures on last MRI differed between groups T1h^−^ and T1h^+^ (*p* = 0.01), there was a considerable overlap of these measures in both groups (see Supplementary Material 2). Therefore, both groups were combined for further analyses.

**Table 1:** Findings in patients without (T1h^−^) and with (T1h^+^) visible T1-hyperintensity of DN

Parameter	T1h^−^	T1h^+^	*p*
Demographic data			
Gender, F/M, *n*	13/4	13/4	1.0 ^1^
Age at time of this study, *mean* ± *SD*, y	48.6 ± 9.5	45.4 ± 10.7	.36 ^2^
EDSS score, median (range)	3.5 (2.0–7.0)	3.5 (1.0–6.5)	.97 ^3^
MS duration *mean* ± *SD*, y	15.8 ± 9.6	16.8 ± 9.5	.76 ^2^
Latency first to last MRI, *mean* ± *SD*, y	10.4 ± 5.1	11.0 ± 3.6	.67 ^2^
Latency last MRI to study, *mean* ± *SD*, m	2.9 ± 3.3	3.0 ± 4.1	.96 ^2^
Number of MRI with linear GBCA, median (range), *n*	4 (2–12)	14 (8–28)	** < .001** ^3^
Cumulative dose of linear GBCA, *mean* ± *SD*, mmol/kg BW	0.63 ± 0.44	1.57 ± 0.62	** < .001** ^2^
Cumulative dose of Gd-DTPA, *mean* ± *SD*, mmol/kg BW	0.40 ± 0.44	1.18 ± 0.57	** < .001** ^2^
Cumulative dose of Gd-DA, *mean* ± *SD*, mmol/kg BW	0.23 ± 0.29	0.39 ± 0.31	.12 ^2^
Laboratory findings			
Serum creatinine, *mean* ± *SD*, µmol/L	70.0 ± 14.3	63.6 ± 9.3	.14 ^2^
Clinical findings			
ICARS total score, median (range)	10 (0–48)	12 (1–44)	.70 ^3^
ICARS static subscore, median (range)	6 (0–26)	3 (0–31)	.78 ^3^
ICARS kinetic subscore, median (range)	7 (0–19)	5 (0–12)	.34 ^3^
ICARS dysarthria subscore, median (range)	0 (0–1)	0 (0–1)	.29 ^3^
ICARS oculomotor movement subscore, median (range)	0.5 (0–4)	2 (0–4)	.60 ^3^
DSST score, median (range)	61 (22–98)	60 (42–95)	.08 ^3^
PASAT-3 *z*-score, median (range)	− 0.4 ([− 1.5]–1.2)	− 0.6 ([− 1.9]–1.2)	.86 ^3^
MRI findings (T1 signal intensity ratio)			
DPSIR on last MRI, *mean* ± *SD*	1.09 ± 0.06	1.16 ± 0.10	**.01** ^2^
DPSIR change from first to last MRI, *mean* ± *SD* ^4^	0.00 ± 0.08	0.09 ± 0.11	**.03** ^2^
GTSIR on last MRI, *mean* ± *SD*	1.07 ± 0.06	1.11 ± 0.05	**.02** ^2^
GTSIR change from first to last MRI, *mean* ± *SD* ^4^	0.04 ± 0.03	0.06 ± 0.04	.27 ^2^
TCS fusion imaging findings (normalized echointensity) ^5^			
DN, *mean* ± *SD*	75.3 ± 23.6	66.7 ± 23.4	.41 ^2^
GPi, *mean* ± *SD*	83.2 ± 20.0	70.8 ± 19.4	.17 ^2^

Abbreviations: *BW* body weight; *EDSS* Expanded Disability Status Scale; *DN* dentate nucleus; *DPSIR* DN-to-pons signal intensity ratio; *DSST* Digit Symbol Substitution Test; *GBCA* gadolinium-based contrast agents; *Gd-DA* gadodiamide (Omniscan®); *Gd-DTPA* gadopentetate dimeglumine (Magnevist®); *GPi* globus pallidus internus; *GTSIR* GPi-to-thalamus signal intensity ratio; *ICARS* International Cooperative Ataxia Rating Scale; *MRI* magnetic resonance imaging; *PASAT-3* 3-s Paced Auditory Serial Addition Test; *TCS* transcranial sonography

^1^ Fisher exact test

^2^*t*-test

^3^Mann-Whitney *U* test

^4^Assessable in 9 T1h^−^ and 14 T1h^+^ patients (for details, see text)

^5^Assessable in 10 T1h^−^ and 11 T1h^+^ patients (for details, see text)

### Cumulative GBCA dose and increase of T1-intensity

**Fig. 1:**
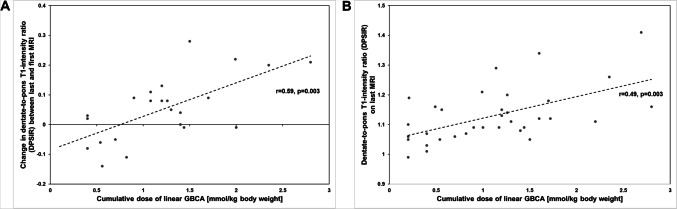
Diagrams showing the relationship between cumulative life-time dose of intravenously administered linear GBCA and dentate-nucleus T1-intensity measures (bilateral mean DPSIR). The *r* and *p* values shown are controlled for potential confounders (age, MS duration, EDSS, latency between first and last MRI). **a** Relationship between cumulative dose of linear GBCA and change of DPSIR in patients with assessable data of first MRI (*n* = 23). **b** Relationship between cumulative dose of linear GBCA and DPSIR on last MRI in all patients (*n* = 34)

Controlling for potential confounders (age, MS duration, EDSS, latency between first and last MRI), DPSIR on last MRI correlated with the cumulative dose of linear GBCA (*n* = 34; *r* = 0.49, *p* = 0.003; Fig. 1a) and of Gd-DTPA (*r* = 0.57, *p* = 0.001) but not Gd-DA (*p* = 0.36). Similarly, controlling for potential confounders, the change in DPSIR over time between first and last MRI correlated with the cumulative dose of linear GBCA (*n* = 23; *r* = 0.59, *p* = 0.003; Fig. 1b) and the cumulative dose of Gd-DTPA (*r* = 0.62, *p* = 0.002) but not Gd-DA (*p* = 0.42). Controlling for potential confounders, no correlation was found between GTSIR on last MRI and the cumulative dose of linear GBCA (*p* = 0.03) nor between the GTSIR change and cumulative dose of linear GBCA (*p* = 0.06).

### Correlation between T1-intensity and echo-intensity

**Fig. 2:**
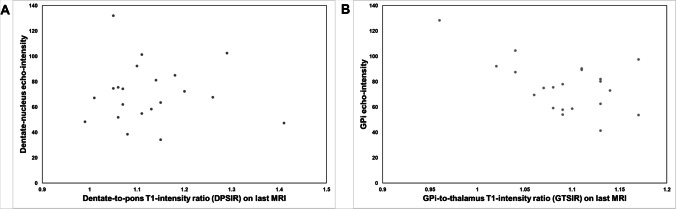
Diagrams showing the relationship between T1-intensity and echo-intensity on digitized analysis of exact real-time MRI-ultrasound fusion images. Note that no correlation between T1-intensity and echo-intensity measures was found. **a** Relationship between dentate-nucleus T1-intensity (bilateral mean DPSIR on last MRI) and echo-intensity. **b** Relationship between globus pallidus internus (GPi) T1-intensity (bilateral mean GTSIR on last MRI) and echo-intensity

TCS images of all 21 patients studied were qualified by the image analysis software to be of adequate quality allowing for the digitized quantification of DN and GPi echo-intensity. No correlation was observed between DPSIR or GTSIR and corresponding DN and GPi echo-intensity measures (each, *p* > 0.14; Fig. 2a, b).

### Correlation between T1-intensity and clinical findings

Latency between last MRI and clinical assessment was 3.0 ± 3.7 months. Controlling for potential confounders (age, MS duration, EDSS, latency between first and last MRI), DPSIR on last MRI correlated with ICARS dysarthria subscore (*n* = 34; *r* = 0.57, *p* = 0.001). Compared to patients without dysarthria (*n* = 30) as assessed on the ICARS, those with dysarthria (*n* = 4) had larger DPSIR on last MRI (median, 1.10 vs 1.27; Mann–Whitney *U* test, *p* = 0.016). DPSIR was unrelated to total ICARS score (*p* = 0.06), ICARS kinetic subscore (*p* = 0.32), ICARS static subscore (*p* = 0.05), ICARS oculomotor subscore (*p* = 0.44), DSST (*p* = 0.32), and PASAT-3 (*p* = 0.20). Controlling for potential confounders, GTSIR was unrelated to any of the clinical test scores.

## Discussion

Data obtained in this study show that multiple administrations of linear GBCAs are related to increased MRI-T1 intensity in the DN (DPSIR). Our findings support the assumption that Gd may accumulate in the DN. However, T1-intensity in the DN and GPi is unrelated to echo-intensity of these structures. Present data suggest a possible association of DPSIR with mild dysarthria but no other change of cerebellar motor or cognitive function.

Our findings are in line with a growing body of evidence showing that multiple intravenous applications of linear GBCAs can lead to an increase of DPSIR independently from a local disruption of the blood–brain barrier [4–19]. As a consequence, the European Medicines Agency and the US Food and Drug Administration issued recommendations to restrict the use of some linear Gd agents used in MRI body scans already in 2017 [2, 11]. A recent retrospective analysis showed significant increase of T1-intensity in the DN even after a single GBCA administration of linear GBCA [34]. If analyzing the relationship between DPSIR increase and cumulative GBCA dose separately for ionic (Gd-DTPA) and non-ionic (Gd-DA) linear GBCA, we could confirm a significant association for Gd-DTPA but not for Gd-DA. This is in contrast to the results of earlier retrospective studies that compared the linear ionic GBCA gadobenate dimeglumine vs Gd-DA [17], and Gd-DTPA vs Gd-DA [18], showing a stronger increase of DN T1-intensity with repeated administration of the non-ionic (Gd-DA) GBCA. This discrepancy may be explained by the relatively low frequency of Gd-DA compared to Gd-DTPA application in our cohort. In addition, an intra-individual interaction between the two GBCA in our cohort may be considered since the majority (73%) of our patients had had received both GBCA. A potentiating effect from prior Gd-DA on subsequent administered ionic GBCA on the increase of DN T1-intensity has been proposed earlier [10].

Increased T1-intensity in deep gray matter structures of MS patients, including the DN and GPi, has been attributed not only to Gd retention but also to abnormal deposition of nonheme iron [35]. Therefore studies were requested to determine whether Gd or iron deposition or their combination is responsible for signal changes in the DN of these patients [35]. TCS can sensitively detect accumulation of trace metals such as iron and associated neurodegenerative changes in deep brain structures in a wide range of brain diseases, including MS [20–22]. Here we aimed at detecting a tissue alteration caused by Gd deposition by applying an elaborate, exact MRI-TCS fusion imaging procedure followed by digitized TCS image analysis. We observed no increase of echo-intensity of DN or GPi, neither visually nor on digitized analysis, and no correlation between T1-intensity and echo-intensity measures. This suggests that Gd retention does neither directly increase tissue echogenicity such as has been shown to occur with iron, copper, or manganese accumulation [20, 22] nor indirectly by secondary neurodegeneration or calcification, which also would increase echo-intensity on TCS [21, 23, 24]. This is in line with studies of human brain tissues and animal studies that consistently failed to demonstrate histopathologic evidence of chronic toxicity arising from retained Gd in neural tissues introduced via intravenous administration [11].

To date, no clear cause-effect relationship has been demonstrated in patients between brain gadolinium exposure and clinical consequences specific to neurological toxicity [36]. Especially, DN T1-hyperintensity was unrelated to the change of disease severity assessed on the EDSS [37]. In an earlier study, however, DN hyperintensity among MS patients was associated with lower verbal fluency [38]. We found an association of DPSIR with the presence of mild dysarthria, in concordance with the previously demonstrated link between DN lesion and dysarthria [39]. In another study, Gd retention-caused alteration of T2-relaxometry measures of DN could be related to reduced information processing speed assessed on the Symbol Digit Modalities Test [15]. We here applied a similar test (DSST) but failed to find a relation between DN T1-intensity and psychomotor speed. Unlike the repeated application of linear GBCAs investigated here, serial injections of more than 20 doses of a macrocyclic GBCA did not evoke newly appearing cerebellar symptoms or signs [40]. This conforms to the idea that linear but not macrocyclic GBCAs may lead to Gd retention in the DN and thereby could possibly influence cerebellar function.

Limitations of this pilot study are the small study sample, the partly retrospective design associated with some variability of MRI acquisition, and the initial classification of cases versus controls based only on the visual presence (vs absence) of DN T1-hyperintensity. The degree of dysarthria was mild in all respective patients and may not necessarily be related to cerebellar dysfunction. Strengths are the strict blinding of raters with respect to clinical and/or imaging data and the high-precision matching of digitally analyzed ROIs on MR and TCS imaging.

In conclusion, present data underpin the view that Gd retention in the brain does not lead to neurodegeneration. Our secondary finding that Gd retention in the DN might possibly cause mild functional disturbance, however, requires confirmation in larger studies employing a detailed assessment of dysarthria subtype and severity.

## Supplementary Information

Below is the link to the electronic supplementary material.

Supplementary file1 (PDF 73 KB)

Supplementary file2 (PDF 73 KB)

## Data Availability

The corresponding author had full access to all the data in the study and takes responsibility for the integrity of the data and the accuracy of the data analysis. De-identified participant data will be shared by request from any qualified investigator. Data sharing requests are made in writing through Dr. Walter and require a formal data sharing agreement with approval from the University of Rostock.

## Code availability

The MATLAB-based customized software tool (B-Mode Assist) used here for digitized ultrasound image analysis is available from Cerebassist.com (for details, see: http://www.cerebassist.com/en/).
