# Supplementary material for: Dentate-nucleus gadolinium deposition on magnetic resonance imaging: ultrasonographic and clinical correlates in multiple sclerosis patients
Source: Neurol Sci. 2021 Nov 4;43(4):2631–9. doi: 10.1007/s10072-021-05702-4 (PMC8918138; doi:10.1007/s10072-021-05702-4)

Supplementary material 1. MRI–ultrasound fusion imaging for digitized echo-intensity analysis of dentate nucleus (DN) in an MS patient with increased T1-intensity of DN.

a) Ultrasound image (semi-coronal transection) of the DN contralateral to transcranial insonation; note that there is no increased echosignal of the DN (arrow; asterisk: fourth ventricle).

b) Reference MRI plane displayed on real-time fusion imaging used to exactly identify the anatomic region of DN on the corresponding ultrasound image shown in (a) and (c). Note that the image contrast in the MRI plane shown is lower than in the original MRI data since the MRI planes were reconstructed by the ultrasound system from the imported MRI DICOM 3D data set.

c) The quadratic section of the ultrasound image representative for DN and surrounding cerebellar parenchyma chosen for offline digitized analysis is highlighted in the left panel. The right panel shows the true-to-scale overlay of MR and semi-transparent ultrasound image.

d) Zoomed section of the ultrasound image shown in (c). For automated measurement of DN echo-intensity, the elliptical marker indicating the region of interest (ROI) with a size of 179 mm^2 was placed manually in the anatomical region of DN. e) Diagram showing the results of digitized echo-intensity analysis in a single patient using the validated software tool.

Online Resource 1

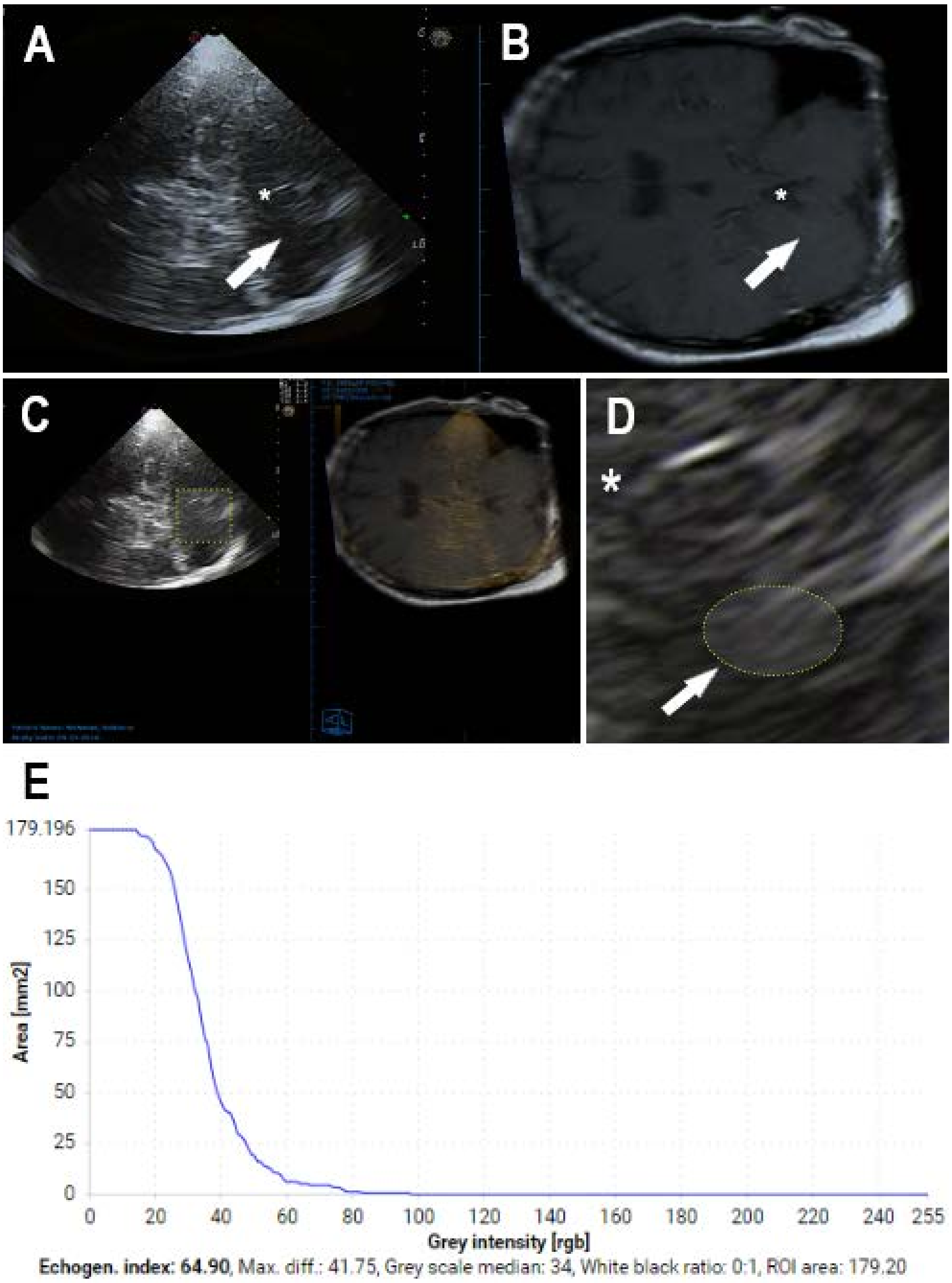

Supplement: Supplementary file 1 — Supplementary file1 (PDF 73 KB) [file 10072_2021_5702_MOESM1_ESM.pdf]
