# Supplementary material for: Dentate-nucleus gadolinium deposition on magnetic resonance imaging: ultrasonographic and clinical correlates in multiple sclerosis patients
Source: Neurol Sci. 2021 Nov 4;43(4):2631–9. doi: 10.1007/s10072-021-05702-4 (PMC8918138; doi:10.1007/s10072-021-05702-4)

Supplementary material 2. Visual grouping of presence versus absence of dentate-nucleus T1-hyperintensity and corresponding objective measures of dentate-nucleus T1-intensity.

The diagram shows the relationship between the visually assessed presence ($Th1^+$) versus absence ($Th1^-$) of dentate-nucleus T1-hyperintensity on at least one of the three most recent MRI scans and objective measures of dentate-nucleus T1-intensity (bilateral average dentate-to-pons-signal intensity ratio on last MRI; bars denote mean ± S.D.). * *t*-test, p=0.01.

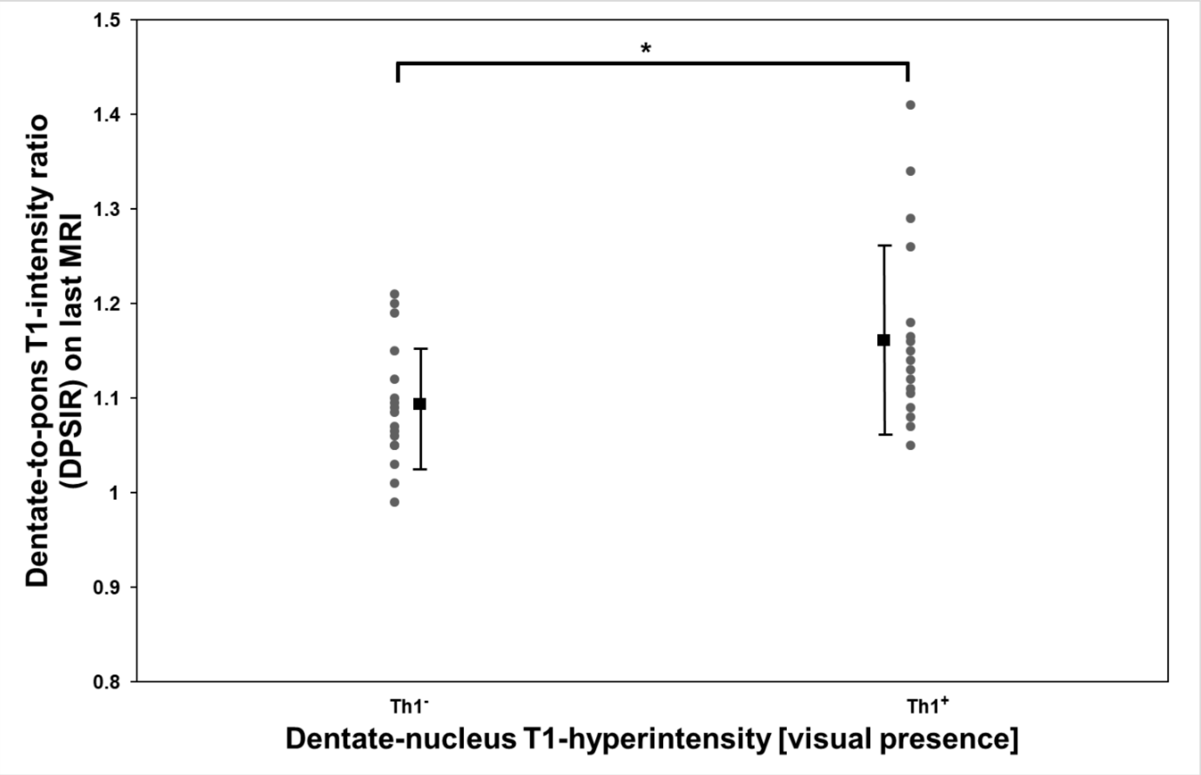

Supplement: Supplementary file 2 — Supplementary file2 (PDF 73 KB) [file 10072_2021_5702_MOESM2_ESM.pdf]
